# Web-Based Remote Control of a Building’s Electrical Power, Green Power Generation and Environmental System Using a Distributive Microcontroller

**DOI:** 10.3390/mi8080241

**Published:** 2017-08-04

**Authors:** Cheng-Yi Chen, Chien-Yuan Liu, Chiu-Chan Kuo, Cheng-Fu Yang

**Affiliations:** 1 Department of Electrical Engineering, Cheng Shiu University, Kaohsiung 83347, Taiwan; k0464@gcloud.csu.edu.tw (C.-Y.C.); ppla32@yahoo.com.tw (C.-C.K.); 2 Department of Computer Science and Information Engineering, Cheng Shiu University, Kaohsiung 83347, Taiwan; 3 Department of Chemical and Materials Engineering, National University of Kaohsiung, Kaohsiung 811, Taiwan

**Keywords:** green power generation, hypertext markup language, smart home, microcontroller

## Abstract

This article proposes a novel, web-based, remote monitoring and control system design for a building’s electrical power, green power generation and environmental system that will save energy. The supervisory control system is based on the use of distributed microcontroller architecture to access programmable logic controllers (PLC) and remote input/output devices through the system hardware framework with uniform Ethernet technology. The programmable logic controller (PLC) can access and control devices directly or through RS-232 and RS-485 serial communication. The distributed microcontroller is the control module designated through an open-source firmware, to transform heterogeneous communication to Modbus transmission control protocol (TCP) communication and to achieve the exchange of information between the host and client controller. The proposed supervisory control and data acquisition (SCADA) system is based on the professional software of InduSoft Web Studio and provides a supervisory control design with a friendly human–machine interface. The system can realize real-time data acquisition and storage, control command transmission, system security and power trend analysis. Finally, the proposed SCADA system can be built directly into the hypertext markup language (HTML) and HTML5 and run on the web server, allowing access from a personal computer or smartphone web browser. Our system goals are to greatly reduce system complexity and maintenance costs with a simple Ethernet architecture. The control system can be easily expanded with the same technology culture outside the restrictive one of the large companies. Hence, this system can easily be used in a smart home system to enhance the quality of its inhabitants.

## 1. Introduction

As the global energy shortage worsens, finding alternative energy sources is becoming an international issue. To meet the requirements of both demand and environmental protection, green energy is the best choice. In tandem with these changes, internet technology has drastically improved the speed and range of human communication and has made work more efficient. Smart products based on the internet have multiplied, enabling major progress in the development of “intelligent living spaces”. In an intelligent living space, various automated home appliances are linked via the internet platform to bring about highly efficient service systems and to ensure the safety and healthiness of the living space. In addition, such systems help provide a more comfortable living environment, heralding a new stage in the evolution of human lifestyle [1].

A Home Energy Management System (HEMS) uses energy micro-sensors in domestic appliances to gather data, via an internet connection, about power consumption and to thereby optimize energy use. Users can monitor their energy consumption status, including location, time, and cost and regulate their personal power consumption profile to achieve energy savings. In addition to saving energy, a HEMS can be joined with a solar energy generation system, enabling the user to meet their own energy needs and sell surplus energy to a utility company. Moreover, all data can be stored in a database system for further system state analysis, thus serving as a reference standard for power management [2,3]. 

Since solar cell technology applications and manufacturing have made rapid progress, the photovoltaic system is very popular for green energy generation. The parallel connection of distributive photovoltaic generation systems and traditional generation units is a world-wide trend. In addition, various strategies are being used to enhance the generation efficiency of solar panels. With the aid of a solar tracking system, solar panels can collect energy more efficiently. Conversion efficiency has also increased. However, to track management and maintenance problems in different fields of generation, the development of a smart monitoring and management system is indispensable [4,5,6].

Recently, with the rapid growth of integrated circuit and electronic communication technologies, embedded systems have become small and affordable. Now, they are widely used in products for both controlling and processing functions. For applications in consumer or industrial products, the demand for automatic process controls cannot be met simply with powerful computer systems because industrial applications must take into consideration both price and performance. Therefore, the demands for microprocessor-driven systems are best met by using automatic systems that incorporate simple embedded microchips at their cores [7]. Currently, due to the rapid advancements in automatic process controls, there is a trend toward an increased use of electronic systems in process control systems. A good communication system is very important for allowing the components of process control systems, such as electronic systems and microcontroller devices, to share information and give the electronic systems of entire manufacturing plants functionalities such as error diagnosis, self-repair and data fusion [8,9]. For many devices, Modbus communication provides effective solutions to the problems of relaying commands and exchanging information. Today, Modbus communication technology is widely used for equipment automation and electronic monitoring in various industries as well as in manufacturing plants. A recent trend with the aim of increasing the performance of a given monitoring system is to choose a uniform communication mode and interface. This helps lower the complexity encountered by the monitoring system when integrating various communication protocols [10], allows the swift integration of master- and client-side monitoring system designs, shortens development cycles, lowers the overall system development cost, and increases the competitiveness of the monitoring system.

In an intelligent building, not only domestic appliances must be included but also mobile apparatuses and multimedia equipment. A future trend, in addition to the provision of diverse options in the host computer system, will be to offer multiple choices for the front-end control interface. The most general one at the moment is the wall-embedded LED, which is connected to the host computer via RS-485 and Ethernet. Users simply issue a command on the control panel display to order the associated equipment to take action. The hand-held microcontroller is another option for remotely sending messages to the control panel via infrared ray, ZigBee, Wi-Fi and so forth, thus offering users significant convenience. Portable appliances such as cellular phones, tablets and laptop computers can all be used to activate various domestic appliances in advance and facilitate a better quality of life [11].

Southern Taiwan is a sunny region, suitable for solar power generation. In the present research, we propose a new concept for a smart building system, using photovoltaic generation and monitoring software and based on a uniform Ethernet distributive module. The generated solar power is used to power a laboratory and is supplemented, when necessary, by power from a utility company. The research staff are able to analyze power consumption details and improve the system’s efficiency, thereby realizing considerable cost savings. The monitoring system uses InduSoft Web Studio [12], which features display and device control, real-time energy consumption and generation data, and power demand data storage. Hence, it helps establish an energy consumption and generation database and offers tools for data inquiry and analysis, enabling us to optimize equipment usage, regulation, and planning. With respect to the proposed hardware system, a personal computer is used as the central control unit and is connected to a distributive microcontroller module through a uniform Ethernet communication. The microcontroller modules are designated through open-source firmware to achieve the exchange of information between host and client controller, to fetch and control the appliance data. This article presents a web-based remote-control system for building and allows the browser, on either a PC or a mobile device, to act as the monitoring and controlling mechanism. Our system is implemented using standardized software, hardware, and distributive microcontroller modules, to deeply reduce system complexity and maintenance costs. Therefore, the control system can be easily expanded in the same technology scenario outside the restrictive one of the large companies. The system was designed, implemented, and gave excellent results in collecting data, transmitting, monitoring, and applying system control. Hence, it will be easy to adapt this system for domestic and factory monitoring applications.

## 2. Experimental System Setup

**Figure 1:**
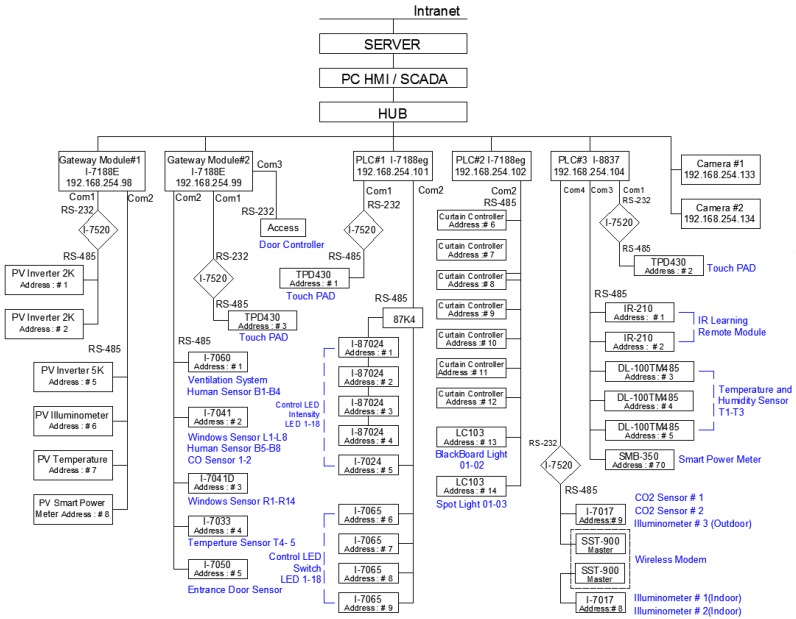
Hardware framework for the experimental system.

Figure 1 shows the proposed experimental hardware framework of the developed system with uniform Ethernet technology. A personal computer with the supervisory control and data acquisition (SCADA) system was designed as the central control unit with a friendly human–machine interface. It possesses two Ethernet ports, one connected to the world-wide network and the other connected to the local intranet network. The former one plays an access route for web server, while the latter stands for the center control application to control the distributive subsystem through transmission control protocol (TCP)/internet protocol (IP) Ethernet. Basically, the host control unit can simply communicate with the distributive control module, from which it can determine the availability of ingredients. Internally, two modes are used to communicate with the associated apparatuses: the first to read/write via PLC communication ports, and the second to use the communication exchange module via the DCON or Modbus RTU communication protocol to directly issue the read/write command to the apparatuses. The detailed functionality of the subsystem in Figure 1 is descripted below.

The solar generation system is composed of two 2 KW fixed type panels and one 5 KW single-axis sunlight tracking texture. The monitoring system makes use of the Modbus TCP communication protocol through the microcontroller module (uPAC-7186EX, 192.168.254.98) and reads the solar panel generation data via a directional meter and inverter. Consequently, the collected data are stored in the system database for real-time generation monitoring and analysis. 

The microcontroller gateway (192.168.254.99) employs virtual communication port technology to map the serial port of the field microcontroller uPAC-7188EX to the serial port of the monitoring system on the host computer. Therefore, the monitoring system can use the DCON communication protocol to access and control measurement results of the remote infrared human body sensor, the CO sensor, the indoor and outdoor thermometers, access control status, window status, and so forth.

Since PLC#1(192.168.254.101) is a palm-size microcontroller, it can control any single output of the 18 analog output channels and adjust the brightness of every individual LED lamp, thus achieving energy savings and dimming functionality. PLC#2 (192.168.254.102) is another palm-size microcontroller, which can control 12 sets of curtain modules and three individual spotlight controls by using the communication protocol of Modbus RTU. Being a compact-size microcontroller, PLC#3 (192.168.254.104) is employed to control two indoor units, an outdoor unit and six fan sets through the IR-210, which is a universal IR learning remote module and, thus, is able to learn IR remote commands for diverse electronic devices. In addition, PLC#3 can pass through RS-485 to fetch the measurements of the I-7017 module, which includes two CO_2_ sensor units and three illumination measuring units. In addition, PLC#3 can acquire power consumption data for each of the circuit loops from the smart power meter SMB-350 (eight three-phase circuit loops) via Modbus RTU communication.

Cameras #1(192.168.254.133) and #2 (192.168.254.134) are internet protocol cameras installed to monitor the entrance and front area of the laboratory, thereby providing a panorama of the laboratory’s access area. The digital images from the cameras are transmitted to the monitoring and control center via TCP/IP. When the entrance gate is opened, the cameras are activated to record photos and videos. The image files are stored in the system for backup and users can check the indoor status via the internet at any time.

## 3. Results and Discussion

**Figure 2:**
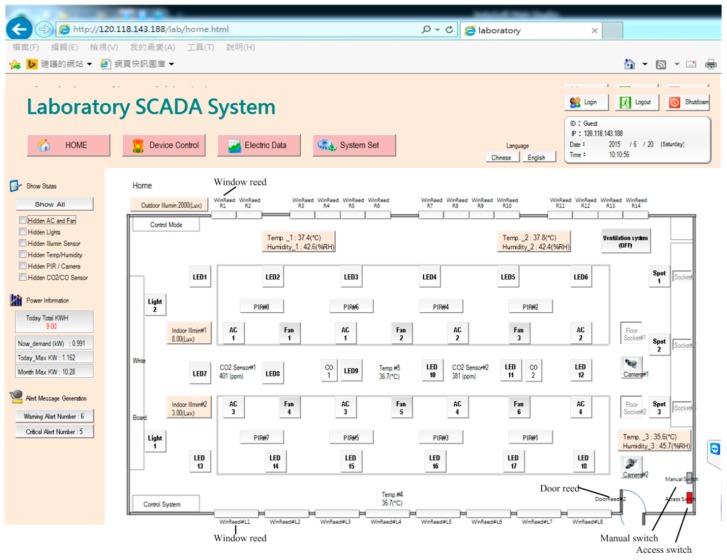
Homepage of the laboratory supervisory control and data acquisition (SCADA) system.

**Figure 3:**
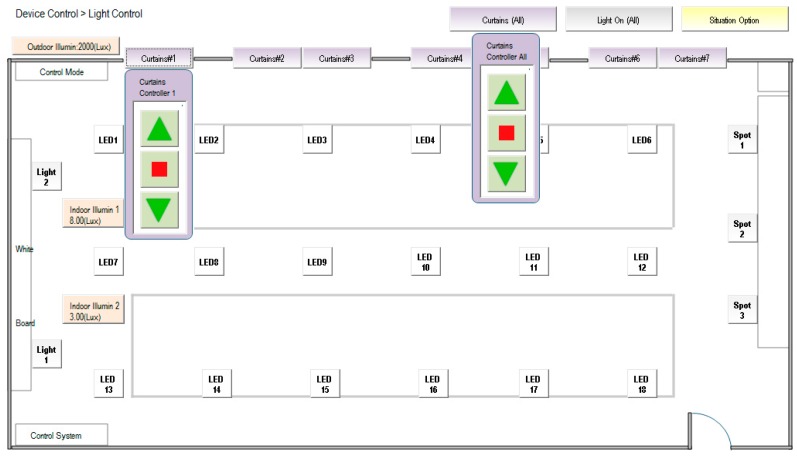
Bookmark for the operation of curtain control.

**Figure 4:**
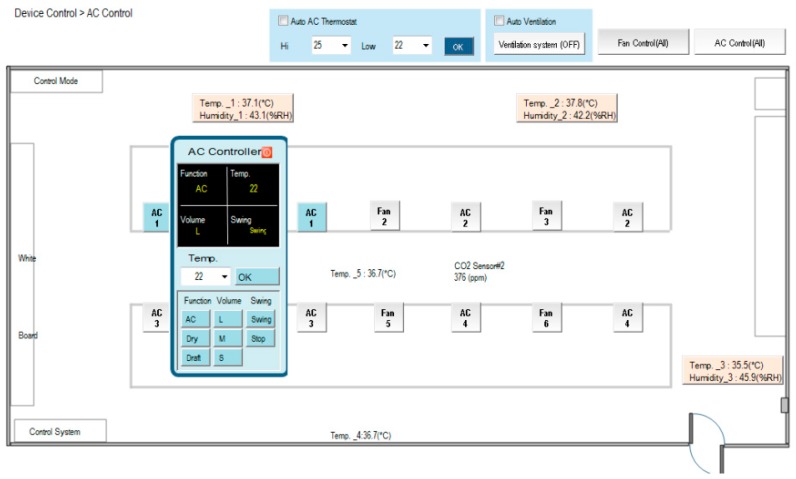
Bookmark for the operation of air conditioning.

**Figure 5:**
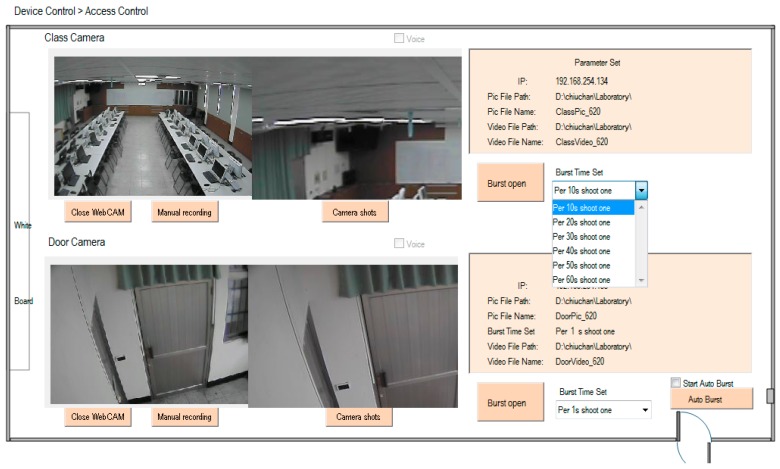
Bookmark for the operation of the camera system.

Figure 2 illustrates the homepage of the human–machine interface of the SCADA system. Ambient status, including characteristics such as illuminance, temperature and humidity, CO_2_ concentration, CO concentration, and personnel access, can be monitored. The automatic illumination control for dimmable, flat LED lighting devices in a building was designed and is described in [13]. Figure 3 shows the operation of curtain control. The electrical power curtains can be controlled synchronously when the curtain (All) button is clicked. Individual curtains can be controlled by clicking on the button for the individual curtain. When the door is opened, a yellow indicator comes on and when a personnel member is detected, a red indicator comes on. CO will activate a blue indicator. Further, if the situation option button is clicked, the lighting status is controlled according to the default setting, which is pre-designed in the embedded controller and allows the SCADA system to activate it. The display panel will also show the proper and comfortable illuminance level, guiding the user to regulate illuminance based on demand. Figure 4 shows the bookmarks for operating air conditioning control. When the air conditioning button is pressed, the corresponding air conditioner is activated and a blue indicator comes on. Simultaneously, the air conditioning control display pops up. As the expected function and its value are clicked and set up, the air conditioner will operate accordingly. Again, when the fan control button is clicked, the fan control display pops up and adjustments can be performed. Figure 5 shows the operational display for the entrance control. When the entrance control button is clicked, a real-time image inside the laboratory can be seen. If the manual record button is clicked, the camera starts recording. Similarly, the camera can take pictures on command. In addition, a time-lapse function is available in this system. It records still images and captures one frame every 10 to 60 s.

**Figure 6:**
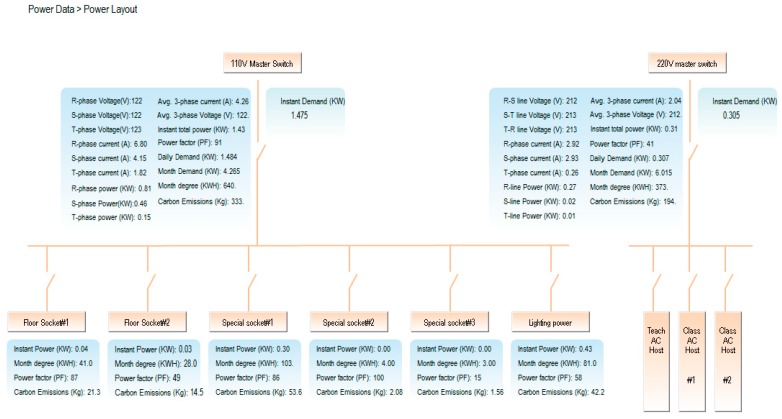
Framework of the power system.

**Figure 7:**
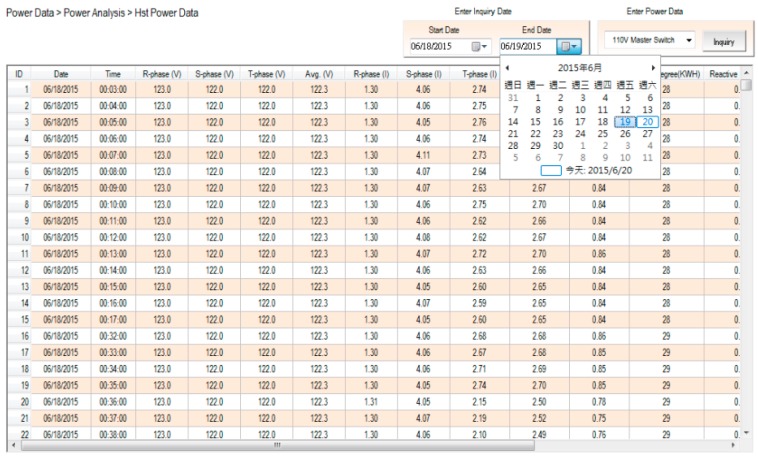
Historical power data query screen.

**Figure 8:**
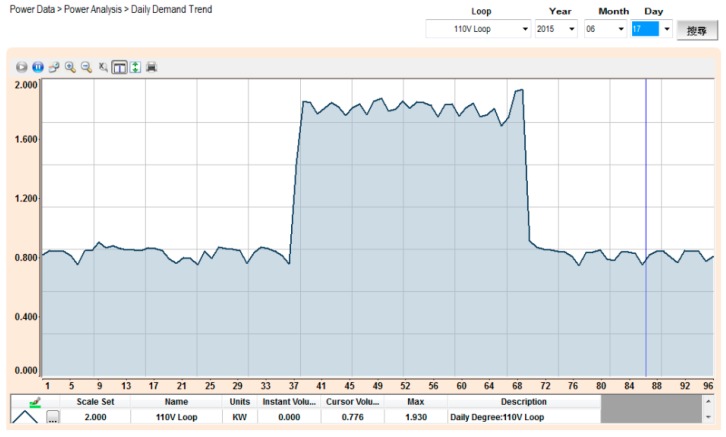
Trend graph of daily power demand.

By using a compact-size microcontroller (PLC), the monitoring system can read the electricity data in the SMB-350, which include the measurement data from the multi-loop meter. The associated quantities in each circuit loop, including voltage, current, real power, power factor, and so forth, will be displayed directly on the page frame of the power system framework, as shown in Figure 6. The power measurement data will be stored in the database as well. Figure 7 shows that the electricity usage data in the database can be received and read easily. Whatever the power data—real-time or historic record—this detailed information can be read by merely inputting the correct time, time-interval, and date. Figure 8 shows the trend graph of the daily power demand of every circuit loop. The trend graph of daily power demand is plotted on a monthly basis, while the trend graph of monthly power demand is plotted on an annual basis. It can also be seen that 96 pieces of power consumption data are gathered each day, and that these reveal detailed time and power usage information. Since the monthly power demand is recorded over one year, the power demand data can provide the user with solid data when negotiating a contract with a utility company. 

**Figure 9:**
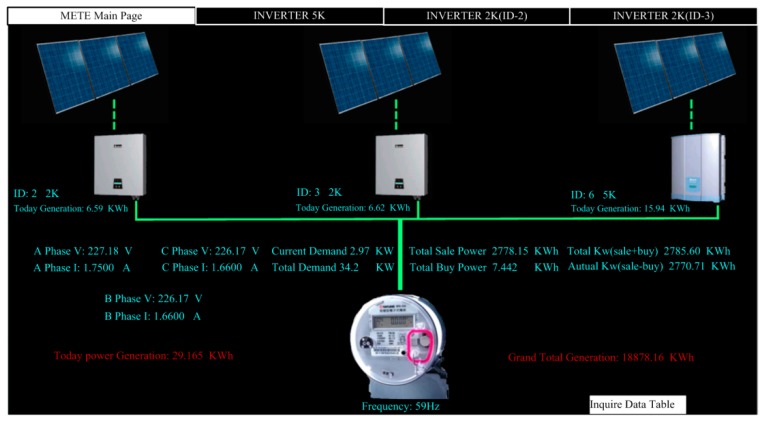
Monitoring page for solar power generation.

**Figure 10:**
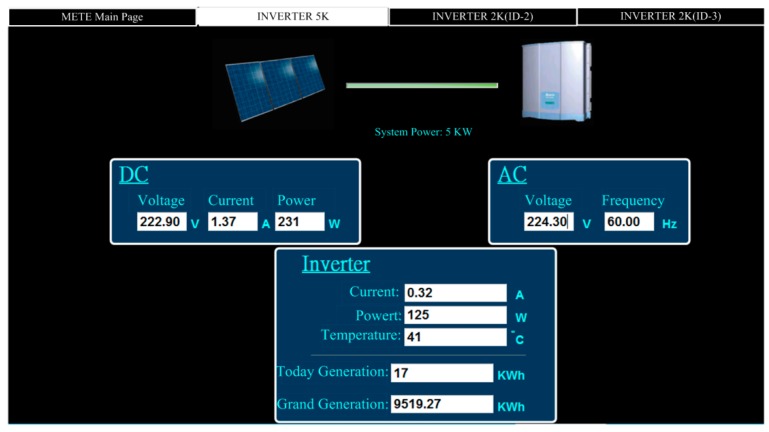
Monitoring page for an individual solar power subsystem.

**Figure 11:**
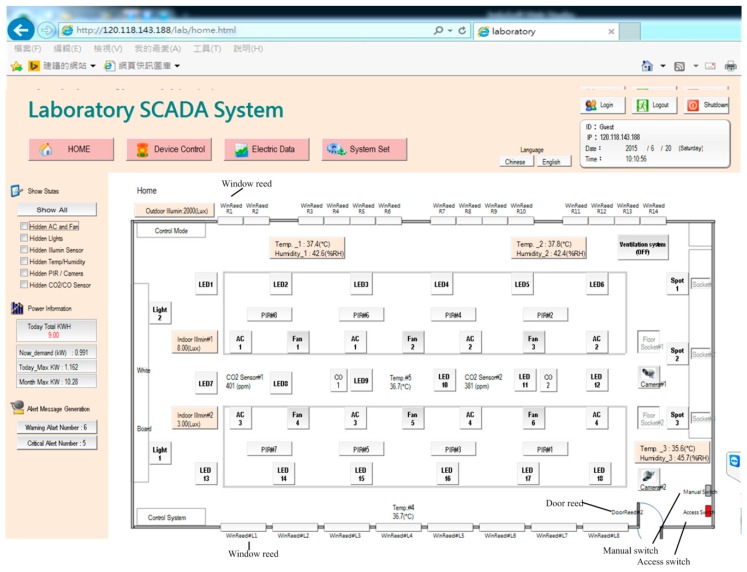
Browser screen from remote connection.

**Figure 12:**
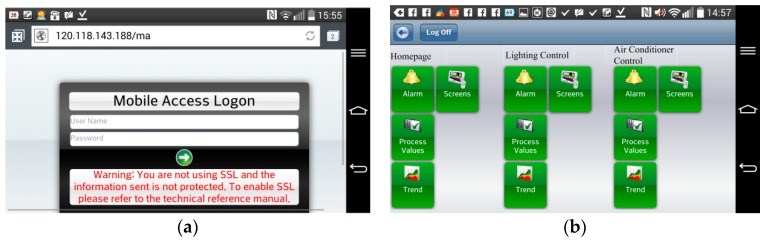
Smartphone accessibility: (**a**) mobile access logon, (**b**) launch page.

**Figure 13:**
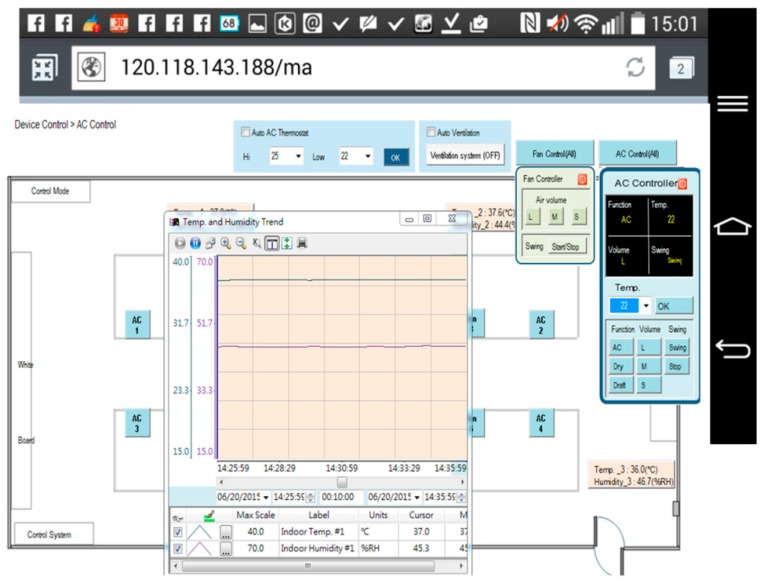
Remote air conditioning monitoring and control using a smartphone.

Figure 9 shows the monitoring page for solar power generation. The first page displays the statuses of total generating capacity, electricity trading, and current power demand. Figure 10 shows the monitoring subpage for an individual inverter, including the generation status and efficiency of that one solar generation unit. Of course, all of the data, for every individual solar generation unit, are stored in the database for further application. Figure 11 illustrates the remote monitoring page from a personal computer via the internet. By using Microsoft Internet Explorer and connecting to the assigned website (http://120.118.143.188/lab/home.html), the system homepage can be reached. An authorized user can remotely monitor and control the apparatus status and set up the system parameters. Figure 12 shows the mobile access login page (http://120.118.143.188/MA) via a smartphone and the corresponding launch system page. Similarly, an authorized user can sign in to monitor and control power usage, lighting, and air conditioning. Figure 13 demonstrates the operation of the air conditioning control through a smartphone or tablet computer and shows the temperature and humidity trends for the indoor environment. 

**Figure 14:**
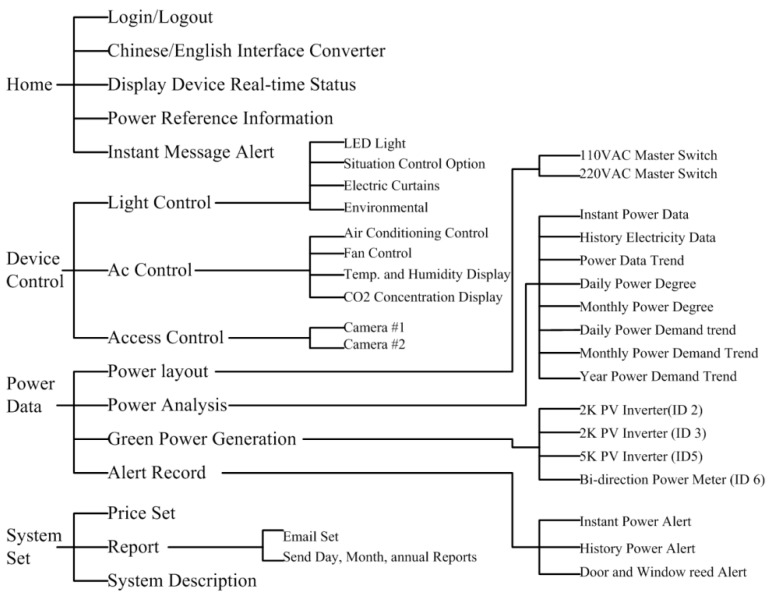
Functional blocks of the proposed system.

Figure 14 demonstrates the functions of the proposed experimental system that have been discussed in this article, which includes the regions of homepage, device control, power data and system configuration. The SCADA control system is established according to the architecture of intranet Ethernet network (see Figure 1). With a user-friendly graphical interface, the proposed design has successfully integrated the controls and measurements of the lighting system, air conditioning system, electric fans, air-interchange system and access system, power system, green power generation, electric window curtains, environment system, system configurations, and so on. With the help of open-source firmware design in uPAC-7188EX, the heterogeneous communication can be transformed to Modbus TCP communication so that the proposed system has a lower maintenance cost and is more flexible for future peripheral expansion. With the quality control of power consumption, the proposed system can also be applied to control the electrical power demand to further save on power consumption expenditure. 

## 4. Conclusions

This research has successfully realized the web-based remote monitoring and control of green power generation, power, and ambient conditions by using Ethernet distributive microcontroller modules. The developed monitoring system features both near-end PC-based control and remote-end web-based control via smartphone and remote PC. In the hardware, commercial and standardized sensor devices and microcontroller modules with open-source firmware are applied to perform distributive and programmable control. This enables the monitoring system to give commands to the subsystem and to fetch the associated data to accomplish monitoring requirements, thus greatly reducing cost and the burden to the system. In addition, this research transforms the heterogeneous communication to Modbus TCP, enhances the diversity of the power equipment applications, and offers system expandability. In the software, the graphic monitoring software design provides users with a friendly graphic operation interface. Through the use of graphic frames, information on lighting, air conditioning and fans, windows and gate contact switches, classroom interiors, and entrance traffic can be read using multiple ambient microsensors. With the collection of the associated data via multiple circuit loops, real-time power consumption information can be acquired. The power consumption data stored in the database can be retrieved as historic data and could help with future planning and adjustments in power equipment installation. This will enhance the laboratory’s power quality and energy efficiency. In addition, by collecting power data from the solar generation unit on the roof and using a pyrheliometer and a modular thermometer, the solar generation capacity and performance ratio can be determined. The novelty of the proposed approach relies on the categorization of different elements into an Ethernet distributive control module, so it is a new conceptual framework to promote the systematic design and implementation of a complicated building automation system involving Modbus communication. This article aims to contribute to fostering the development of a web-based remote control of a building system with uniform Ethernet technology, acting as a user resource for both practitioners and researchers in the design of integrating sensors, actuators, securities, and controllers needed to develop their activities. This integration of software, hardware, and control mechanisms has created a successful example of a smart building, which can easily be adjusted for use in factories and domestic buildings. In the future, home energy management will be studied by optimal analysis approaches as the data collected are rich enough.
